# Array comparative genomic hybridization: Results from an adult population with drug-resistant epilepsy and co-morbidities

**DOI:** 10.1016/j.ejmg.2011.12.011

**Published:** 2012-05

**Authors:** Elizabeth C. Galizia, Maithili Srikantha, Rodger Palmer, Jonathan J. Waters, Nicholas Lench, Caroline Mackie Ogilvie, Dalia Kasperavičiūtė, Lina Nashef, Sanjay M. Sisodiya

**Affiliations:** a Department of Clinical and Experimental Epilepsy, UCL Institute of Neurology, Queen Square, London, WCIN 3BG, United Kingdom; b Department of Neurology, King’s College Hospital, London, SE5 9RS, United Kingdom; c North East Thames Regional Genetics Service, Great Ormond Street Hospital for Children NHS Trust, London, WC1N 3BH, United Kingdom; d Cytogenetics Laboratory, Guy’s and St Thomas’ NHS Foundation Trust, London, SE1 9RT, United Kingdom

**Keywords:** Array comparative genomic hybridization, Copy number variation, DNA, Epilepsy, Co-morbidity

## Abstract

**Background:**

The emergence of array comparative genomic hybridization (array CGH) as a diagnostic tool in molecular genetics has facilitated recognition of microdeletions and microduplications as risk factors for both generalised and focal epilepsies. Furthermore, there is evidence that some microdeletions/duplications, such as the 15q13.3 deletion predispose to a range of neuropsychiatric disorders, including intellectual disability (ID), autism, schizophrenia and epilepsy.

We hypothesised that array CGH would reveal relevant findings in an adult patient group with epilepsy and complex phenotypes.

**Methods:**

82 patients (54 from the National Hospital for Neurology and Neurosurgery and 28 from King’s College Hospital) with drug-resistant epilepsy and co-morbidities had array CGH. Separate clinicians ordered array CGH and separate platforms were used at the two sites.

**Results:**

In the two independent groups we identified copy number variants judged to be of pathogenic significance in 13.5% (7/52) and 20% (5/25) respectively, noting that slightly different selection criteria were used, giving an overall yield of 15.6%. Sixty-nine variants of unknown significance were also identified in the group from the National Hospital for Neurology and Neurosurgery and 5 from the King’s College Hospital patient group.

**Conclusion:**

We conclude that array CGH be considered an important investigation in adults with complicated epilepsy and, at least at present for selected patients, should join the diagnostic repertoire of clinical history and examination, neuroimaging, electroencephalography and other indicated investigations in generating a more complete formulation of an individual’s epilepsy.

## Highlights

► Copy number variants may predispose to a range of neuropsychiatric disorders. ► Array CGH may reveal relevant findings in adults with complex phenotypes. ► Likely pathogenic copy number changes were identified with a yield of 15.6%. ► Array CGH should join the diagnostic repertoire for adults with complex phenotypes.

## 1 Introduction

The field of molecular genetics has evolved rapidly, with array comparative genomic hybridization (array CGH) established as a diagnostic tool in molecular genetics [1]. Array CGH provides whole genome analysis capable of detecting genomic rearrangements just a few kilobases in size, depending on the platform used[2]. By comparison, conventional karyotyping has been reported as in some cases being able to detect deletions as small as 5 Mb and duplications as small as 2 Mb [3]. In idiopathic intellectual disability (ID), array CGH increases the yield by roughly an additional 10% compared to conventional karyotyping alone [4].

The application of array CGH to investigate patients with epilepsy has led to the recognition of recurrent pathogenic microdeletions, with 15q13.3, 16p13.11 and 15q11.2 microdeletions emerging as significant risk factors for generalised epilepsies, as well as focal epilepsies in the case of 16p13.11 [5–7]. Furthermore, there is evidence that some microdeletions/duplications predispose to a range of neuropsychiatric disorders: for example, the 15q13.3 deletion that has been associated with intellectual disability (ID), autism, schizophrenia and epilepsy [8].

There are limited published data on the use of array CGH in adults with unexplained complex neuropsychiatric phenotypes. Often adults represent a small proportion of most cohorts, with only one study investigating a purely adult patient group with idiopathic ID (mean age 35 years) [9]. With improved paediatric care, more patients with complex phenotypes or unexplained ID have survived into adulthood [10]. These adults would not have had access to current diagnostic tools during their paediatric follow up, when they were most likely to undergo genetic testing. For patients who have previously undergone genetic testing, the limitations of the methods used then, means that the genetic diagnosis may not have been identified [10]. When a chromosomal abnormality has been identified previously, the diagnosis may remain unchallenged, even when the clinical picture does not correlate with reports in the literature. With new, more accurate methods re-evaluation of such cases is warranted.

With the growing evidence that sub-microscopic chromosomal abnormalities may be causal or act as risk factors for a number of neuropsychiatric disorders, including epilepsy, we hypothesised that array CGH would reveal relevant findings in a group of adult patients with epilepsy and complex phenotypes. We report results for two independent adult groups, demonstrating the clinical usefulness of array CGH and the important role molecular cytogenetics has to play in establishing a syndromic diagnosis.

## 2 Methods

### 2.1 Ethics

The work at the National Hospital of Neurology and Neurosurgery (NHNN) was approved by the relevant local Research Ethics Committee. Patient consent, or assent from the family, was obtained following appropriate counselling for those patients in whom array CGH was performed as part of their clinical diagnostic work up, or as part of an ongoing research project into the role of genetics in epilepsy. Data from King’s College Hospital NHS Foundation Trust (KCH) were part of an audit into the utility of this investigation in one clinician’s clinic and not part of a research project. Patients were appropriately counselled as part of routine clinical practise when genetic testing was performed.

### 2.2 Cases

54 patients with drug-resistant epilepsy followed up at or referred for assessment to the NHNN and 28 patients followed up at or referred to KCH underwent array CGH.

Selection at NHNN was based on the presence of epilepsy in combination with one or more of the following characteristics, determined from medical records and outlined in Table 1: (1) developmental delay/intellectual disability, as determined by formal neuropsychometric testing or clinical assessment/contemporary documentation of developmental delay; (2) dysmorphism; (3) family history of epilepsy, neuropsychiatric disorder or learning disability, as defined by the presence of at least one affected first or second degree relative; (4) personal history of a psychiatric disorder; (5) other co-morbidities (including developmental anomalies, abnormal neuroimaging, migraine). These criteria were selected based on the evidence for a common genetic basis for developmental and neuropsychiatric disorders[8], with array CGH performed in cases seen over an 18 month period between 2009 and 2010. Epilepsy was classified according to the International League Against Epilepsy (ILAE) Commission on Classification and Terminology, 2005–2009.

All cases that had array CGH performed at KCH, who had this investigation recommended between September 10 2009 and August 17 2010, were included in the audit, a period during which the test was offered based on similar criteria to those listed for the NHNN cohort but excluding abnormal neuroimaging or migraine. These criteria were: a history of epilepsy of unknown aetiology associated with any of the following a) developmental delay, b) learning disability, c) dysmorphism; d) mental health problems, including autistic spectrum disorders, and/or e) a family history of the same.

### 2.3 Array CGH platform and analysis

Array CGH analysis of NHNN patients was performed by the North East Thames Regional Genetics Service, using the NimbleGen 12 × 135 K, whole genome v3.0 array chip, according to the manufacturer’s instructions. This platform has 135,000 oligonucleotide probes distributed across the genome with a probe separation of approximately 13 kb. Pooled reference DNA was obtained commercially (Promega, UK and Kreatech, the Netherlands). The signal intensity plots were analysed using CGH Fusion^®^ software (InfoQuant, UK). The software average log-2-ratio threshold was set at 0.35, and a minimum of 3 consecutive probes was required for a positive call by the software, giving this platform a functional resolution of 200 kb (i.e. a 95% probability of picking up every variant over 200 kb; smaller variants are also detected, but with a lower probability)[2]. Whilst these settings can lead to a number of false positive calls, they reduce the risk of filtering out regions of copy number change that may prove relevant to the case being investigated. All calls were then checked manually to ensure they fulfilled the criteria for copy number change. During this manual check, a more stringent criterion was applied in order to limit the number of false positive calls. To meet this criterion, any call made by the software had to include at least three consecutive probes which passed a log-2 ratio threshold of 0.4. Calls not meeting this additional criterion were removed from further consideration. The data from 2/54 cases were considered to be of too poor quality to be able to distinguish between false positive and true calls with confidence. These were removed from further analysis.

King’s College Hospital array CGH was performed by the Guy’s and St Thomas’ NHS Foundation Trust South East Thames Regional Cytogenetics Laboratory as part of their clinical diagnostic service, using an Agilent custom platform comprising approximately 44,000 probes across the genome, as previously described[11]. Signal intensity plots were analysed using Agilent’s DNA analytics software, with a 3 probe sliding window providing a mean detection interval of 200 kb. Individual patient DNA samples were compared with individual DNA from patients without epilepsy, rather than against pooled commercial reference DNA as was the case for the NHNN group.

### 2.4 Classifying significance of copy number variants

**Fig. 1:**
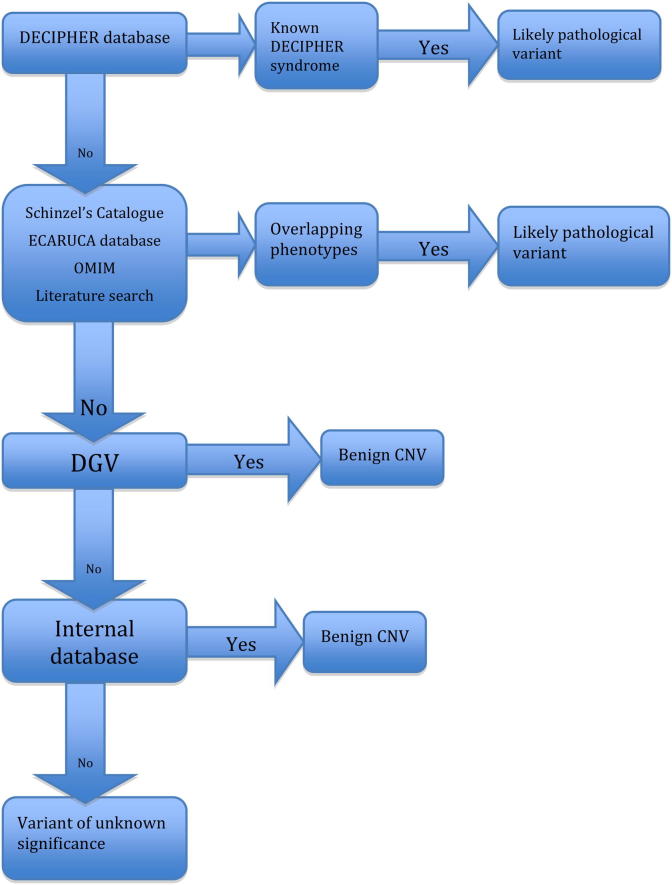
Flow diagram outlining the process used for identifying those variants of likely pathological significance, benign variants unlikely to be contributing to the clinical phenotype and variants of unknown significance. ECARUCA = European Cytogeneticists Association Register of Unbalanced Chromosome aberrations; OMIM = online Mendelian inheritance in Man; DGV = database of Genomic variation; CNV = copy number variant.

We generated a filter to categorize variants into three groups: likely pathogenic, benign and unknown significance (see Fig. 1). This filter is adapted from the algorithm proposed by Buysee et al.[12], acknowledging that methods for determining the significance of copy number variants (CNVs) are still in evolution.

For the NHNN group, first we identified variants that overlapped with known deletion or duplication syndromes using the DECIPHER database (https://decipher.sanger.ac.uk). We consulted Schinzel’s Catalogue of Unbalanced Chromosome Aberrations in Man, 2nd edition, and the ECARUCA database (http://www.ecaruca.net/), to identify those variants that overlapped with previously described variants, comparing the reported phenotypes with those of our patients. Genes encompassed by variants were identified using the UCSC genome browser (http://genome.ucsc.edu; NCBI build 36/hg18). Phenotypic correlations were made through the publicly available databases, OMIM and the published literature. By finding overlapping CNVs in patients with similar phenotypes to those already reported, we identified variants of likely pathogenic significance.

Where no correlation could be made between a CNV and a defined syndrome or disease state, we searched the Database of Genomic Variants (DGV) (http://projects.tcag.ca/variation/) to identify those variants reported in control populations. Complete overlap by a minimum of two controls with the same type of variant (i.e. gain or loss) in this database was required for the variant to be deemed a benign CNV. Where there was overlap with just one control in DGV, or controls with a different variant to the one being considered, the variant was then compared to a locally compiled track. This track is based on 300 anonymised samples, collated internally and shown, following comparison with DGV, investigation of gene content and comparison of patient phenotypes with those known to be associated with the genes involved, not to include any variants of known pathological significance. If overlap was noted between the CNV under consideration and the locally compiled track, the variant was assigned to the group of benign CNVs.

All regions that did not fulfil any of the above criteria at the end of the filter process were classified as variants of unknown significance.

Among the NHNN patients, microarray was performed in both parents for 5 patients, FISH alone was performed for the parents of 2 other patients and in one case, the patient’s mother and twin brother underwent FISH, but the father was not available for testing. When parental studies were available, we identified those variants arising de novo and those inherited from a normal or phenotypically similar parent.

Results for the KCH cohort were reported by the Guy’s Cytogenetic laboratory as follows: normal (only with variants recognised in control populations), showing CNVs of likely pathogenic significance, and showing CNVs of uncertain significance. Established population polymorphisms were defined where the DGV showed at least three publications indicating the presence of the imbalance in control populations. Likely pathogenic significance was assigned to imbalances corresponding to established microdeletion regions or susceptibility loci, those containing genes with known function related to the referral indication, and large imbalances (> ∼5 Mb). Inheritance studies were recommended to help assess these findings further. The KCH group included three patients related to a case previously diagnosed in the same laboratory as having a 15q13.3 microdeletion.

## 3 Results

52 NHNN patients (27 male, aged 18–81 years) were included in the final analysis; patient characteristics are given in Table 1. Twenty-two patients were recorded as being ‘dysmorphic’. Thirty-three patients had ID/DD. Neuropsychometric testing was not available in one NHNN patient, as there was no clinical indication. Neuroimaging abnormalities were reported in 55.8%; details are given in Table 1.

In the NHNN group, we identified seven variants of likely pathological significance in 52 patients, giving a yield of 13.5%. Table 2a outlines the variants found. Two had a chromosomal abnormality diagnosed in childhood, with no further details available. One further case was also previously documented as having a chromosomal abnormality, not confirmed by repeat karyotype or array CGH.

In the KCH group, there were 3 individuals with a 15q13.3 microdeletion, tested during the audit period, who were related to another known case and have therefore been excluded from analysis. There were 25 remaining patients (17 male): four had one pathogenic variant each, whilst one had two pathogenic variants, giving a yield of 20% (see Table 2b).

### 3.1 Regions of unknown significance

We identified 65 variants of unknown significance in the 52 cases within the NHNN group, with an average size of 523 kb and average gene content of 2.4 genes; in the KCH cohort, 5 variants of unknown significance in 25 cases were identified, with an average size of 219 kb and average gene content of 3 genes (see Supplementary Table). The difference in the number of CNVs of unknown significance between the two patient groups is most likely due to the higher probe density of the array used to test the NHNN group.

Determining the effect these variants may have on the clinical phenotype is often difficult [12], particularly when inheritance is unknown, a common problem in our cohort as many parents were unavailable for testing.

Examples of such variants include two CNVs, one gain and one loss, involving *CHRNA7*. In Case 3, apart from the 3.6 Mb deletion at 9p24.3p24.2, there was a 360 kb gain involving *CHRNA7*. Neuropsychiatric abnormalities have been described in six out of 11 patients with microduplication of *CHRNA7* and in family members also found to have the same microduplication[13]. Only one case out of the 11 described had seizures. There was no formal neuropsychiatric history in our patient. In another patient, a small region of copy number loss disrupting *CHRNA7* was also identified. No regions of likely pathogenic significance were identified in this particular case. Deletion of *CHRNA7* has been implicated in idiopathic generalised epilepsy (IGE) in individuals with 15q13.3 deletion syndrome [14, 15]. Work by Shinawi et al. (2009) [16] supports the hypothesis that haploinsufficiency of *CHRNA7* is largely responsible for the phenotype associated with 15q13.3 deletion; however, the deletion detected in our patient is smaller than that reported by Shinawi et al., (203 kb vs 680 kb) and there are a number of differences in the phenotypes they describe and that of our patient. Our patient had periventricular heterotopia and syringomyelia, with documented complex partial and secondarily generalised tonic clonic seizures, no intellectual disability, seizure onset recorded at age 30year and no relevant family history. We cannot comment further on the effect the small region of copy number loss noted may have in this case and the possible interaction with the congenital brain malformation.

## 4 Discussion

In two independent groups of adults with epilepsy and co-morbidities, we identified copy number variants judged to be of pathogenic significance in 13.5% (7/52) and 20% (5/25) respectively, noting that different criteria and microarray platforms were used, giving an overall yield of 15.6%, in keeping with the literature [1].

These patients represent the ‘lost generation’, people who, if seen as children today, would probably have array CGH as a first line investigation. Our results are comparable to the average diagnostic yield of 10–30% in published studies of array CGH in epilepsy [17, 18] and in idiopathic developmental delay/intellectual disability (DD/ID) with or without associated congenital anomalies[4], suggesting that a putative genetic cause or contribution may be important in a significant proportion of selected patients with epilepsy of unknown cause.

For many genetic disorders described in children there is often a paucity of information about the evolution of the disorders with age[10]. Adult patients may therefore present with features not recognised as part of an otherwise well-described syndrome, so that a genetic diagnosis is less likely to be suspected. Furthermore, early history may have become vague or lost. During adulthood, patients may acquire other diseases, gain weight and, particularly in epilepsy, acquire head or facial injuries that further obscure the phenotype. Specialists treating adults in the many varied clinical fields are usually not trained dysmorphologists or clinical geneticists. As a consequence, there may be less awareness of, or appreciation for, other co-morbidities, that may point to and be explained by a unifying genetic diagnosis. The presence of a family history or significance of co-morbidities may not be appreciated if history taking focuses only on one feature, for example epilepsy, dismissing others that form part of a complex phenotype. For these reasons patients may not be referred to a clinical geneticist. Limited access to clinical geneticists and their expertise may be perceived barriers for appropriate referrals. Establishing the diagnosis brings an end to the ‘investigative odyssey’ and aids counselling of patients and their families in terms of prognosis, treatment options and risks for future generations [19–21]. Our findings also suggest that such testing is indicated, in the right setting, even when imaging reveals a structural abnormality that might be considered the cause.

Phenotypic heterogeneity makes phenotype-genotype correlations challenging or impracticable, as highlighted by deletions of 15q13.3 or 16p13.11[20]. Incomplete penetrance is often suggested as a reason for variations in phenotype associated with a given CNV. Girirajan and Eichler [22] suggest variable expressivity as being the likely explanation for clinical heterogeneity and propose a 2-hit model, after the finding that ∼25% of cases with one deletion, studied using a custom array, also carried another large deletion or duplication. They propose that the interaction of the two CNVs is more likely to predispose to a severe phenotype. In Case 3 (Table 2a), for example, we identified a region of copy number change of unknown significance affecting the *CHRNA7* gene; an MRI brain scan also showed widespread longstanding, possibly perinatal, ischaemic changes. We cannot determine from available data whether the *CHRNA7* duplication or the structural changes (or both or neither) might act as a ‘second hit(s)’. As the resolution of genomic methods and experience in the field grow, it is likely both that more putatively pathogenic variants will emerge and that fewer will be deemed of ’unknown significance’.

Determining the significance of CNVs and how they should be reported is a challenge. The presence of CNVs within ‘disease-free’ populations is well documented [23, 24], whilst inherited variants cannot be assumed to be benign [25]. Algorithms for determining the significance of CNVs have been proposed by Koolen et al. [26], and Edelmann and Hirschhorn [19]. These methods have been employed by most studies investigating use of array CGH in idiopathic ID. Typically CNVs, either inherited from a normal parent or found in control populations are identified as ‘benign’ variants. Such variants are deemed unlikely to contribute to the patient’s phenotype and are removed from further consideration early on in the algorithms. The remaining variants are then assigned to one of the two remaining groups, likely pathogenic or variants of unknown significance. However such an approach could exclude variants that may contribute to the clinical picture [12]. Buysee et al. [12], propose an alternate decision tree, which we adapted, as a filter for the categorization of variants into the three groups of likely pathogenic, benign and unknown significance (see Fig. 1).

There is a recognised need for the standardization of interpreting and reporting CNVs. Keeping within the constraints of our filter may have led to some variants being classified here as of unknown significance, whilst in a clinical laboratory setting they may be regarded as probably benign. Whilst laboratories may adopt similar approaches for determining the clinical significance of a CNV, the final interpretation has been shown to be quite variable [27]. Publicly available databases for control populations, as used in our filters for both groups, aid in identifying those variants that are unlikely to contribute to a disease state. However, even in these databases phenotypic data for the controls are typically not available and, furthermore, one cannot assume the individuals do not carry variants that predispose to latent, later onset (after recruitment) or recessive disease [12, 4]. This has led to calls for the sharing of data and development of publicly available databases for genotype and phenotype data that would aid in establishing the role of CNVs, particularly rare CNVs, currently classified as of unknown significance [28, 4]. Detailed gene-driven phenotyping may improve understanding of CNVs of unknown significance, or clarify the full phenotype associated with a given, putatively pathogenic, CNV [21].

A number of different platforms are available for detection of CNVs, varying in their resolution (from ∼1 Mb for BAC arrays to a few kilobases for oligonucleotide and single nucleotide polymorphism arrays), coverage of the genome (targeted vs whole genome coverage) and source of comparator DNA. Depending on the array used, whole genome arrays alone may miss clinically relevant regions [29] and balanced translocations are typically not detected [4].

Our patient groups represent a highly selected population. Much of the work using whole genome microarray in idiopathic DD/ID relates to selected patient cohorts, with only two small studies investigating the use of whole genome microarray in unselected cases [30, 31]. The yield from microarray as a first line test in an unselected cohort of consecutive DD/ID patients has been inferred as being ∼19% [11, 32]. In epilepsy, array CGH has been used to identify candidate regions likely to harbour epilepsy-related genes [17], with recurrent microdeletions at 15q13.3, 16p13.11 and 15q11.2 later being shown to cumulatively account for ∼3% of cases with generalised epilepsy [5–7], making them the three most common recurrent CNVs in epilepsy. Mefford et al. [6], and Heinzen et al. [7], also identified a number of other rare CNVs.

From a practical perspective the question arises as to which patients would benefit from array CGH, as there is little guidance about patient selection in the adult population. Increasingly, cytogenetics laboratories are introducing array CGH as a first line test in place of karyotyping. However, there is still a need for appropriate selection in patient groups such as ours, where the link between isolated neurocognitive disorders and copy number variation has not yet been clearly demonstrated. We selected patients based on the presence of certain individual or familial co-morbidities. De Vries et al. [33], proposed a scoring system that has been adopted by a number of groups investigating the use of array CGH in DD/ID. The aim of the scoring system is to identify those individuals more likely to have a deletion/duplication, by attributing points according to the presence of a number of features including pre-natal and post-natal growth abnormalities, dysmorphism and family history of intellectual disability. De Vries et al. [33], demonstrated that those individuals with pathogenic CNVs had higher scores than those without. The inclusion of severity of intellectual disability and the presence of abnormal neuroimaging was suggested by Engels et al. [34], to further refine the de Vries score. We were unable to apply the proposed scoring system to the two patient groups we describe here due to lack of detail available in the clinical notes, particularly regarding facial and non-facial dysmorphism, pre-natal growth abnormalities and degree of ID. This is likely to reflect the situation in most adult specialist clinics. However, the evidence would suggest that the more severe the phenotype overall, the more likely it is that a genomic abnormality will be identified [1].

We conclude that array CGH be considered an important investigation in adults with complicated epilepsy and should join the diagnostic repertoire of clinical history and examination, neuroimaging, electroencephalography and other indicated investigations. Only further data will enable its final position in diagnosis to be established, and consideration will be needed of the management of potential ‘incidental’ findings of variants relating to other conditions, though in some cases, any such ‘other’ conditions and epilepsy may be part of the same genomic disease [21].

## Competing Interests

None declared.

## Funding

This work was partly undertaken at UCLH/UCL, which received a proportion of funding from the Department of Health’s NIHR Biomedical Research Centres funding scheme. We acknowledge funding support from The Freemasons’ Grand Charity, The Arthur James Paterson Charitable Trust, Wellcome Trust (Grant 084730; SMS), National Institute for Health Research (08-08–SCC; SMS), CLRN FSF Grant CEL1300 (SMS), and The Katy Baggott Foundation.

## Figures and Tables

**Table 1:** Characteristics of the patient group from the National Hospital for Neurology and Neurosurgery.

Characteristic	Percentage (Numbers)
Gender	
Male	51.9% (27)
Female	48%(25)

Epilepsy classification	
Focal	75%(39)
Primary Generalised	3.8%(2)
Unclassified	19.2%(10)
Mixed	1.9%(1)

Facial Dysmorphism	
Yes	42.3%(22)
Features defined	36.3%(8)
Features not defined	63.7%(14)
No	55.7%(29)
Not documented	1.9%(1)

Developmental Delay/Intellectual Disability (DD/ID)	
Present	63.4%(33)
Absent	34.6%(18)
Not documented	1.9%(1)
Psychiatric Disorder	
Yes	36.5%(19)
No	63.5%(33)

Co-morbidities	
Yes	71.1%(37)
No	28.8%(15)

History of Febrile Seizures	
Yes	11.5%(6)
No	88.5%(46)

Family History of Epilepsy/ID/DD/Psychiatric disorder	
Yes	51.9%(27)
No	42.3%(22)
Unknown	5.8%(3)

Neuroimaging	
Normal	32.7%(17)
Abnormal	55.8%(29)
Cerebellar atrophy	6.9%(2)
Generalised Atrophy	13.8%(4)
Hippocampal sclerosis	10.3%(3)
Dual pathology^a^	3.4%(1)
Congenital malformation^b^	37.9%(11)
Meningioma	3.4%(1)
High signal lesions^c^	13.8%(4)
Ischaemic perinatal insult	3.4%(1)
Hydrocephalus	3.4%(1)
Intrinsic lesion with dysplastic features	3.4%(1)
Not done	10%(5)
Unknown	1.9%(1)

a Hippocampal sclerosis and a cortical malformation.

b Congenital malformations included agenesis of the corpus callosum, subependymal nodular heterotopia and dysembryoplastic neuroepithelial tumour.

c Four patients with high signal lesions; these were reported non-specific in two, of ischaemic origin in one and related to previous head injuries in the last.

**Table 2a:** Cases from the National Hospital for Neurology and Neurosurgery with variants deemed likely pathogenic.

Case No.	Variant Cytoband	Copy number change type	Size (kb)	Genes^a^	Karyotype	Epilepsy^b^ (seizure type)	Co-morbidity	Inheritance
1	15q11.2q13.1	Deletion	5356.7	SPG6; NDN; SNRPN; UBE3A; ANC; MKRN3; MAGEL2; AK124131; BC034815; C15orf2; IPW; PAR1; ATP10A; GABRA5; GABRG3.	Normal	Partial, of unknown cause (Atonic; GTCS; CPS)	short stature; spastic quadriparesis; dysconjugate gaze; insomnia.	unknown
2	1p36.33p36.32	Deletion	2333.6	GABRD; SKI; PEX10; TNFRSF18; TNFRSF4; SCNN1D; CPSF3L; TAS1R3; DVL1; AKIP; VWA1; ATAD3B; MRPL20; CDC2L1; MIB2; MMP23B; MMP23A; GNB1; NAD; CALML6; PLCH2; HES5; TNFRSF1; PRDM16.	NA	Unclassified (GTCS; Atonic)	dysmorphic; congenital hydrocephalus; spina bifida occulta; microcephaly; obstructive sleep apnoea.	de novo
3	9p24.3p24.2	Deletion	3644.9	DOCK8; ANKRD15; DMRT1; VLDLR; SMARCA2; RFX3; GLIS3; HLA-HA8; KCNV2.	NA	Presumed Partial, attributable to perinatal insult (Undefined)	microcephaly; dysmorphic face; shortened 5th metacarpals; family history of intellectual disability; extensive ischaemic brain damage.	unknown
4	15q11.1q13.1	Duplication	8369.1	POTEB; TUBGCP5; CYFIP1; NIPA1; NIPA2; NDN; SNRPN; UBE3A; ATP10A; GABRB3; GABRA5; GABRG3; OCA2; HERC2.	Chromosome 15 abnormality - No details available	Partial, of unknown cause (Atonic; GTCS; CPS)	floppy-baby; autism; behavioural problems; developmental delay.	de novo
5	16p13.11	Deletion	946.8	NDE1; MYH11; ABCC6.	NA	Partial, attributable to malformation of cortical development (CPS; GTCS; Tonic seizures)	premature requiring ventilatory assistance; panhypopituitarism; partial agenesis of corpus callosum, subependymal nodular heterotopia; valvular heart disease.	unknown
6	6q22.31q22.33	Deletion	4060	NKAIN2; STL; TPD52L1; HEY2; NCOA7; HINT3; C6orf17; RSPO3; PTPRK.	Normal	Unclassified (GTCS; Absences; Myoclonic jerks)	low average IQ; myopia; lymphoedema; multinodular goitre; macular degeneration; dysmorphism; microcephaly; family history of epilepsy.	maternal
7	4p16.3p12	Duplication	48570	ADRA2C; DOK7; SH3BP2; FGFR3; IDUA; PDE6B; HTT; LBN; ADD1; MSX1; WFS1; SLC2A9; DRD5; QDPR; MCDR2; PMX2B; PARK5; APBB2; CNGA1.	Chromosome 4 trisomy	Unclassified (GTCS)	cataracts; kyphoscoliosis; dysmorphism; seizures associated with recurrent respiratory arrest.	unknown

a OMIM associated genes identified using UCSC genome browser (http://genome.ucsc.edu/).

b Epilepsy syndrome based on the revised terminology and concepts for organisation of seizures and epilepsies: Report of the ILAE Commission on Classification and Terminology, 2005–2009 NA = not applicable.

**Table 2b:** Variants for patients from King’s College Hospital deemed likely pathogenic.

Case No.	Array karyotype	Copy number	Size (kb)	Genes^a^	Inheritance
1	15q13.2q13.3	x1	1.981 Mb	MIR211; TRPM1; KLF13; OTUD7A; CHRNA7; ARHGAP11A	unknown
2	Xp22.33q28 16p11.2	mosaic x1 x1	whole chromosome 525 kb	mosaic Turner syndrome SPN; QPRT; MAZ; C16orf53; MVP; KCTD13; TAOK2; DOC2A; GSD12; PPP4C; TBX6; YPEL3; MAPK3; CORO1A	maternal
3	15q11.2q13.2(19,109,124–28,153,416)x2∼4, 15q13.2q13.3(28,910,478–30,226,235)x2∼3	mosaic x2/x3/x4	10.3 Mb	BCL8; TUBGCP5; CYFIP1; NIPA2; SPG6; MKRN3; MAGEL2; NDN; C15orf2; SNRPN; PAR5; PWCR1; PAR1; SNORD115; ANCR; ATP10A; GABRB3; GABRA5; GABRG3; OCA2; HERC2; APBA2; NDNL2; TJP1; TRPM1; KLF13; OTUD7A; CHRNA7	unknown
4	16p13.11	x1	1.144 Mb	RRN3; NDE1; MYH11; ABCC1; ABCC6	unknown
5	7q35	x0	908 kb	CTNAP2	unknown

a OMIM associated genes identified using UCSC genome browser (http://genome.ucsc.edu/).
