# Supplementary material for: Array comparative genomic hybridization: Results from an adult population with drug-resistant epilepsy and co-morbidities
Source: Eur J Med Genet. 2012 May;55(5-3):342–8. doi: 10.1016/j.ejmg.2011.12.011 (PMC3526772; doi:10.1016/j.ejmg.2011.12.011)
Supplement: Supplementary file 1 [file mmc1.doc]

| **Case Number** | **Cytoband** | **Break points** | **Variant type (loss vs gain)** | **Inheritance** | **Size(kb)** | **Gene content** |
| --- | --- | --- | --- | --- | --- | --- |
| 3 | Yq11.223q11.23 | 23092110-26861840 | loss | Unknown | 3796.7 | TTTY17A; BPY2; DAZ1; DAZ2; PRY; CDY1; GOLGA2LY1; TTTY4;BPY2; DAZ3; DAZ2;  TTTY3;PRY2 |
|  | 15q13.3 | 29843280-30204470 | gain | Unknown | 361.1 | CHRNA7 |
|  |  |  |  |  |  |  |
| 6 | 8p23.1 | 6837430-7884930 | loss | Unknown | 1047 | DEFA1; DEFA3; DEFA5;  DEFB3; SPAG11; DEFB4 |
|  | 10q11.22 | 47461580-47804260 | loss | Unknown | 342.6 | no genes |
|  | 10q11.22 | 48464500-49059130 | loss | Unknown | 594.6 |  |
|  | 10p15.1 | 4875240-5110770 | loss | Unknown | 235.5 | AKR1C2 |
|  |  |  |  |  |  |  |
| 7 | 7p12.1 | 52496960-52674420 | loss | Unknown | 177.5 | no genes |
|  | 8p21.2 | 24159150-24269050 | loss | Unknown | 109.9 | ADAM28 |
|  |  |  |  |  |  |  |
| 8 | Yq11.223 | 22044550-22481510 | loss | Unknown | 436.9 | RBMY1A1 |
|  |  |  |  |  |  |  |
| 9 | Xp21.2p21.1 | 31076070-31560020 | gain | Unknown | 483.9 | DMD |
|  |  |  |  |  |  |  |
| 10 | 6q12 | 67038340-67436300 | loss | Unknown | 397.9 | no genes |
|  |  |  |  |  |  |  |
| 11 | 19p13.3 | 36349-235800 | loss | Unknown | 199.4 | OR4F17; PPAP2C |
|  |  |  |  |  |  |  |
| 12 | 10q21.1 | 56555600-57162870 | loss | Unknown | 607.2 | no genes |
|  |  |  |  |  |  |  |
| 13 | 8p23.1 | 11337340-11507510 | gain | Unknown | 170.1 | FAM167A; BLK |
|  | 2p13.3p13.2 | 72398250-72909670 | loss | Unknown | 511.4 | EXOC6B |
|  |  |  |  |  |  |  |
| 14 | 2p24.3 | 15163470-15725850 | gain | Unknown | 562.3 | NBAS;DDX1 |
|  | 21q21.3 | 29432230-29611960 | loss | Unknown | 179.7 | C21orf7; BACH1 |
|  |  |  |  |  |  |  |
| 15 | Yq11.22.3q11.23 | 23165090-26878990 | loss | Unknown | 3712.9 | TTTY17A; BPY2; DAZ1; DAZ2; PRY; CDY1; GOLGA2LY1; TTTY4; BPY2; DAZ3; DAZ2;  TTTY3;PRY2 |
|  |  |  |  |  |  |  |
|  |  |  |  |  |  |  |
| 16 | 2q22.3 | 146325610-147163420 | loss | Unknown | 837.8 | no genes |
|  |  |  |  |  |  |  |
| 17 | 7q31.33 | 125692150-126000320 | gain | paternal | 308.1 | GRM8 |
|  |  |  |  |  |  |  |
| 18 | 17q21.31q21.32 | 41755950-42121250 | loss | Unknown | 365.3 | NSF |
|  | Xq26.3 | 134382920-134665830 | gain | Unknown | 282.9 | no genes |
|  | 2p16.3 | 51157800-51328140 | loss | Unknown | 170.3 | no genes |
|  |  |  |  |  |  |  |
| 19 | 8p23.1 | 6840764-7880533 | loss | Unknown | 1039.7 | DEFA1; DEFA3; DEFA5;  DEFB3; SPAG11;DEFB4 |
|  |  |  |  |  |  |  |
| 20 | 4q28.3 | 137431930-137991455 | loss | Unknown | 559.5 | no genes |
|  | 8p23.1 | 6837430-7828410 | gain | Unknown | 990.9 | DEFA1; DEFA3; DEFA5;  DEFB3; SPAG11;DEFB4 |
|  |  |  |  |  |  |  |
| 21 | 1p36.11 | 25384690-25610360 | loss | Unknown | 225.6 | SYF2; RHCE; SMP1; RHD |
|  |  |  |  |  |  |  |
| 22 | 2q23.2 | 149881540-150029780 | gain | Unknown | 148.2 | no genes |
|  |  |  |  |  |  |  |
| 23 | 7q35 | 143504898-143705123 | gain | Unknown | 200.2 | CTAGE4; ARHGEF5 |
|  | Xp21.3 | 27967090-28145570 | loss | Unknown | 178.5 | no genes |
|  | Xq28 | 153064830-153165610 | gain | Unknown | 100 | OPN1LW; OPN1MW; TEX28 |
|  |  |  |  |  |  |  |
| 24 | 5q13.2 | 68416570-69980950 | loss | Unknown | 1564.4 | SLC30A5; CCNB1; CENPH; MRPS36; CDK7; TAF9; RAD17; MARVELD2;OCLN; SERF1A; SMN2; GTF2H2 |
|  |  |  |  |  |  |  |
| 25 | 1p36.11 | 25507260-25610360 | loss | Unknown | 103.1 | RHCE; SMP1; RHD |
|  | Xp22.33 | 3727150-3863410 | gain | Unknown | 136.2 | no genes |
|  |  |  |  |  |  |  |
| 26 | 1p36.11 | 25458050-25637160 | loss | Unknown | 179.1 | RHCE; SMP1; RHD |
|  | 5q13.2 | 68879570-70690780 | loss | Unknown | 1811.2 | OCLN; SERF1A; SMN2; NAIP;GTF2H2B |
|  | 15q13.3 | 30204470-30407660 | loss | Unknown | 203.1 | CHRNA7 |
|  | 21q22.3 | 46797770-46907080 | gain | Unknown | 109.3 | DIP2; S100B;HRMT1L1 |
|  | 7q31.31 | 119946240-120118730 | loss | Unknown | 172.4 | KCND2 |
|  |  |  |  |  |  |  |
| 27 | 8p23.1 | 7228100-7821154 | loss | Unknown | 593 | DEFB3; SPAG11; DEFB4 |
|  |  |  |  |  |  |  |
| 28 | 1p36.11 | 25458050-25616500 | loss | Unknown | 158.4 | RHD; SMP1; RHCE |
|  |  |  |  |  |  |  |
| 29 | 18q21.1 | 42537460-42656480 | gain | Unknown | 119 | SIAT8E; PIAS2 |
|  |  |  |  |  |  |  |
|  |  |  |  |  |  |  |
| 30 | 1p21.1 | 103839805-104032630 | loss | Unknown | 192.8 | AMY2B; AMY2A; AMY1C |
|  | 7q22.1 | 101904922-102125709 | gain | Unknown | 220.7 | CAPRI; POLR2J2; |
|  | 14q11.2 | 20391611-20494386 | gain | Unknown | 102.7 | RNASE3 |
|  |  |  |  |  |  |  |
| 31 | 1p36.11 | 25458050-25616500 | loss | Unknown | 158.4 | RHD; SMP1; RHCE |
|  | 8p22 | 13642220-13819680 | gain | Unknown | 177.4 | no genes |
|  | 12q24.32 | 125700690-126134930 | gain | Unknown | 434.2 | no genes |
|  |  |  |  |  |  |  |
| 32 | 2q11.2 | 96860700-97454600 | gain | Unknown | 593.9 | no genes |
|  | 10p15.3p15.2 | 2686830-3183330 | gain | Unknown | 496.5 | PFKP |
|  | Xp22.33 | 11150-279370 | gain | Unknown | 268.2 | PGPL; PPP2R3B |
|  |  |  |  |  |  |  |
| 33 | 16p11.2 | 28740930-28854360 | loss | Unknown | 113.4 | ATXN2L; TUFM; SH2B1;  ATP2A1; RABEP2; CD19 |
|  | 17q21.31q21.32 | 41755950-42121250 | loss | Unknown | 365.3 | NSF |
|  | 1q21.1 | 144111250-144519240 | gain | Unknown | 407.9 | HFE2; TXNIP; RBM8A; GNRHR2;PEX11B; ITGA10; CD160; PDZK1; GPR89A |
|  |  |  |  |  |  |  |
| 34 | 5q13.2 | 70367240-70704810 | loss | Unknown | 337.5 | GTF2H2; OCLN; NAIP |
|  | 8p23.1 | 7192510-7884930 | loss | Unknown | 692.4 | DEFB3; SPAG11;  DEFB4; |
|  | 14q11.1q11.2 | 18543620-19124600 | gain | Unknown | 580.9 | POTEG |
|  |  |  |  |  |  |  |
| 35 | 8p23.1 | 6837430-7884930 | gain | Unknown | 1047.5 | DEFA1; DEFA3; DEFA5;  SPAG11B; DEFB4 |
|  | 9p24.3 | 199250-320800 | gain | Unknown | 121.5 | DOCK8 |
|  |  |  |  |  |  |  |
| 36 | 8p23.1 | 7217180-7884930 | loss | Unknown | 667.7 | DEFA3; SPAG11B; DEFB4 |
|  |  |  |  |  |  |  |
| 37 | 6q26 | 162771740-162885460 | loss | Unknown | 113.7 | PARK2 |
|  |  |  |  |  |  |  |
| 38 | 14q11.1q11.2 | 18543620-19359930 | gain | Unknown | 816 | POTEG |
|  | 11p12 | 36554370-37478760 | gain | Unknown | 924.3 | RAG2 |
|  |  |  |  |  |  |  |
| 39 | Xq26.3 | 134682750-134857720 | loss | Unknown | 174.9 | CT45A1; CT45A4;CT45A5; SAGE |
