# Supplementary material for: Array comparative genomic hybridization: Results from an adult population with drug-resistant epilepsy and co-morbidities
Source: Eur J Med Genet. 2012 May;55(5-3):342–8. doi: 10.1016/j.ejmg.2011.12.011 (PMC3526772; doi:10.1016/j.ejmg.2011.12.011)
Supplement: Supplementary file 2 [file mmc2.doc]

| **Case Number** | **Cytoband** | **Break points** | **Copy Number** | **Inheritance** | **Size (kb)** | **Gene Content** |
| --- | --- | --- | --- | --- | --- | --- |
| 6 | Xq13.2 | 73,356,089 -73,595,528 | x2 | Maternal | 239 | SLC16A2; MIR545; MIR374B; MIR374A; NCRNA00182; ZCCHC13 |
| 7 | 17q12 | 32,720,427-32,887,854 | x3 | Unknown | 167 | ACACA; C17orf78; TADA2A |
| 8 | 19q13.41 | 58,334,343-58,334,762 | x1 | Unknown | 0.419 | ZNF347 |
| 9 | 1p22.1  5p13.3 | 92,137,811 -92,367,878  32,146,491-32,185,480 | x3  x4 | Maternal  Unknown | 230  39 | BRDT; EPHX4; BTBD8  GOLPH3 |
